# Investigating in-vehicle distracting activities and crash risks for young drivers using structural equation modeling

**DOI:** 10.1371/journal.pone.0235325

**Published:** 2020-07-02

**Authors:** Khaled Shaaban, Sherif Gaweesh, Mohamed M. Ahmed

**Affiliations:** 1 Department of Civil Engineering, Qatar University, Doha, Qatar; 2 Qatar Transportation and Traffic Safety Center, Qatar University, Doha, Qatar; 3 Department of Civil and Architectural Engineering, University of Wyoming, Laramie, Wyoming, United States of America; 4 Turner-Fairbank Highway Research Center, United States Department of Transportation, McLean, Virginia, United States of America; Tongii University, CHINA

## Abstract

Distracted driving has been considered one of the main reasons for traffic crashes in recent times, especially among young drivers. The objectives of this study were to identify the distracting activities in which young drivers engage, assess the most distracting ones based on their experiences, and investigate the factors that might increase crash risk. The data were collected through a self-report questionnaire. Most participants reported frequent cell phone use while driving. Other reported activities include adjusting audio devices, chatting with passengers, smoking, eating, and drinking. A structural equation model was constructed to identify the latent variables that have a significant influence on crash risk. The analysis showed that in-vehicle distractions had a high effect on the crash likelihood. The results also indicated that dangerous driving behavior had a direct effect on the crash risk probability, as well as on the rash driving latent variables. The results provide insight into distracted driving behavior among young drivers and can be useful in developing enforcement and educational strategies to reduce this type of behavior.

## Introduction

Traffic crashes account for a significant number of serious injuries and deaths worldwide. Young drivers are responsible for a disproportionately large number of these crashes [1–6]. Not using seat belts, drink-driving, speeding, fatigue, and distracted driving are some of the leading causes of traffic crashes [7, 8]. The National Highway Safety Administration estimates that distracted driving is the reason for approximately 10% of all fatal crashes in the United States [9]. Distracted driving refers to engaging in an activity that distracts attention while driving. In-vehicle distractions can be visual, manual, or cognitive. In case of visual distraction, the driver takes his or her eyes off the road. Manual distraction occurs when a driver takes his or her hands off the steering wheel. With respect to cognitive distraction, the driver takes his or her mind off driving. Typical distracting activities that drivers engage in while driving include pulling down a window, setting up side mirrors, looking away from the roadway, dialing a cell phone, responding to a ringing cell phone, text messaging, adjusting radio/CD, music listening, and getting lost in thought [10–12]. More than one type of distraction can occur at the same time.

Despite the different types of distractions, the research has tended to focus more on cell phone related distractions. For instance, various studies have shown that using cell phones while driving is associated with reduced driving performance, increased driver reaction times, reduced control of the vehicle, and a higher risk of a crash [13–23]. Nevertheless, cell phone use is responsible for approximately 15 to 25% of all distraction-related fatal crashes, and almost three-quarters of all drivers are distracted by other types of behaviors [9, 24]. Therefore, more comprehensive investigations are needed.

Young drivers are involved in more distraction-related crashes than any other age group [25–27]. Moreover, numerous studies have reported higher rates of texting while driving among young drivers than other age groups [14, 28–34]. In general, young drivers exhibit such behaviors either because they are not aware [35–37] or despite being aware that they affect their driving performance [38, 39].

Qatar is a developing country in the Arabian Gulf region, where young drivers are involved in a significant proportion of all traffic crashes. In Qatar, the minimum driving age is 18, and it is illegal to use a cell phone while driving. In general, traffic laws and enforcement are very similar to those in Western countries [40]. Drivers aged 18 to 25 years are involved in more traffic crashes than any other age group. For instance, young drivers accounted for 32.6% of all fatalities, 29.3% of major injuries, and 26.9% of minor injuries in 2011 [41]. In general, driver behavior in Qatar is considered aggressive [41–45]. Few studies have investigated distracted driving in Qatar and in the entire region, which also includes Bahrain, Kuwait, Saudi Arabia, Oman, and the United Arab Emirates.

The purpose of this study was to conduct a survey using a detailed self-report questionnaire to identify and assess the different types and levels of distracted driving among young drivers in Qatar. The study also aimed to determine the most distracting activities based on young drivers’ experiences and the factors that might increase crash risk. This study is one of the first attempts to investigate distracted driving in this region. Based on the findings, the study also proposed possible solutions to the distracted driving problem to help policy makers improve traffic safety in the Arabian Gulf region.

## Materials and methods

### Survey design

A survey questionnaire was designed to obtain young drivers’ perceptions of the most distracting activities while driving and assess their frequency. The target population was young drivers in Qatar, who had a valid driver’s license. In this study, a young driver was defined as a driver aged between 18 and 25 years. The survey form included different possible causes of distracted driving in addition to a separate column for additional responses if the participants’ response did not correlate with any of the reasons provided. The form was prepared in English and Arabic languages to give a chance to different nationalities living in Qatar to answer the questions.

The questionnaire included several sections. The first section included questions regarding the demographics of the participants, including gender, age, highest level of education, and working status. The second section was related to their driving experience. It included questions regarding years of driving experience, kilometers traveled by car per month, means of the daily commute, and the number of days per week they commute by car. The third section covered questions related to traffic crashes and violations. For traffic crashes, the participants were asked if they were involved in any traffic crashes since they obtained their driving license, the total number of traffic crashes, and the severity of the last crash. For traffic violations, the participants were asked to provide the number of received violations since they obtained their driving license. Respondents who did not receive any violations skipped this section.

The fourth section addressed speeding and cell phone use. It included questions related to the rate of speed while driving within the city and the reasons for rash driving, if applicable. It also included questions about the use of cell phones. As part of the survey, participants were asked about situations when they would make a phone call or text while driving. The fifth section collected information regarding their driving habits. The habits were classified into four groups (safe habits while driving, disruptive habits inside the car, driving habits towards other drivers, and behaviors of other people on the road). The frequency of repeating these habits was captured using a 5-point Likert scale (never, rarely, sometimes, often, and always).

In the sixth section, the drivers reported their risk perception and risky behavior. Four statements were constructed, and the respondents marked their level of agreement using a 5-point Likert scale (strongly disagree to strongly agree). The two risk perception statements included being concerned regarding the high probability of being involved in a major traffic crash and being concerned regarding driving a dangerous environment. The two risky behavior statements included their acceptance of exceeding the speed limit to get ahead of traffic or when the weather conditions are good, and the traffic police are not present. Finally, the last section explored the opinion of the participants regarding multiple proposed solutions for the problem. The survey has been granted a research ethics exemption since the participants were anonymous, and their responses were not linked to their personal identifications.

The survey form was distributed to drivers between the age of 18 to 25 with a valid Qatari driver’s license in public places, including malls, libraries, universities, and sports clubs. The interviews were presented as an opportunity to make a difference in saving the lives of young drivers in Qatar. The participants were conveniently selected because of budget constraints. The sample was collected in a way to achieve a representative sample according to gender. A total of 450 questionnaires were distributed and collected. Only 401 questionnaires were considered complete and available for the analysis. The remaining questionnaires had a high percentage of missing responses, and hence they were not used in the analysis. All data were entered into a spreadsheet database. Team members, who were not involved in the data entry process, verified the data for data accuracy. The verification for accuracy was achieved by comparing each survey form against the data entered.

### Descriptive statistics

For the survey responses collected, the count, percentage, mean, and standard deviation (SD) are provided in Table 1. The gender distribution of the sample resembled the population in Qatar, with 75% males and 25% females. Most of the participants (60.3%) had a diploma, and 32.7% had a high school diploma.

**Table 1:** Sample descriptive analysis.

No.	Variable	Categories	Count / Percentage	Mean	SD
	***Demographics***				
Q1	Gender	Male (1)	301 / 75.1%	1.25	0.433
		Female (2)	100 / 24.9%		
Q2	Age	Young Drivers (18 to 25)	401/ 100.0%	20.81	1.880
Q3	Level of Education	Primary (1)	2 / 0.5%	3.71	0.606
		Preparatory (2)	2 / 0.5%		
		High School Diploma (3)	131 / 32.7%		
		Diploma (4)	242 / 60.3%		
		University Degree or Higher (5)	24 / 5.97%		
Q4	Working status	Working (1)	170 / 33.83%	1.62	0.575
		Student (2)	212 / 61.69%		
		Employed and Studying at Same Time (3)	19 / 4.48%		
		Without Work and Not Studying (4)	0 / 0.0%		
	***Driving Experience***				
Q5	Years of driving experience	Discrete Variable (0 to 7)		2.43	1.799
Q6	Average kilometers driven/month	1000 or less (1)	163 / 40.6%	2.13	1.14
		1001 to 2000 (2)	94 / 23.4%		
		2001 to 3000 (3)	72 / 18.0%		
		More than 3000 (4)	72 / 18.0%		
Q7	Mean of Transportation	Car (1)	380/ 94.8%	1.08	0.36
		Taxi (2)	10 / 2.5%		
		Bus (3)	11 / 2.7%		
		Motorcycle (4)	0 / 0.0%		
		Other (5)	0 / 0.0%		
Q8	Car Use Per Week	Daily (1)	223 / 55.6%	1.93	1.28
		4–5 Days (2)	73 / 18.2%		
		2–3 Days (3)	44 / 11.0%		
		One Day (4)	31 / 7.7%		
		Never Use It (5)	30 / 7.5%		
	***Crash involvement and Violations***				
Q9	Crash Involvement	Yes (1)	230 / 57.4%	1.43	0.495
		No (2)	171 / 42.6%		
Q10	No. of Traffic Crashes	Discrete Variable (1 to >10)		1.98	1.48
Q11	Last Encountered Crash Severity	Fatal (1)	0 / 0.00%	2.86	0.37
		Injury (2)	30 / 16.9%		
		PDO (3)	148 / 83.1%		
Q12	Traffic Violations	Yes (1)	184 / 45.9%	1.54	0.50
		No (2)	217 / 54.1%		
Q13	No. of Traffic Violations	Discrete Variable (1 to >10)		1.90	1.25

Regarding the driving experience of participants, the average number of years of driving for the participants was 2.43 years. The monthly average mileage was 2,000 km or less for 64.0% of the respondents. The survey showed that 94.8% of the respondents used the car as their main means of transportation, which indicated that they depend more on their private cars than on public transportation. Moreover, a high percentage of the respondents, 55.6%, used the car every day during the week.

With respect to the crash involvement and violations, more than 57.4% of the participants reported being involved in at least one crash. The average number of crashes for the participants was 1.98, with a standard deviation of 1.48. Most of the encountered crashes were property damage only crashes (PDO) with a percentage of 83.1%, and 16.9% of the crashes involved injuries. A high percentage of the participants, 45.9%, indicated that they committed at least one traffic violation. The average number of violations was 1.9, with a standard deviation of 1.25.

The participants’ perception of different proposed solutions to improve the sense of safe driving was also collected. Six potential solutions were provided and ranked using a five-point Likert scale (very poor to very useful). Table 2 shows the results of the descriptive statistics for the proposed solutions. Revoking the license of frequent violators had the highest average score (3.22), followed by an award system for good drivers without violations (3.08), then increase or toughening the punishment or fine for violators (3.00).

**Table 2:** Descriptive statistics for the proposed solutions.

No.	Variable	Description	Mean	SD
Q33A	Increase the presence of traffic police and enforcement	1 → Very poor	2.97	1.37
Q33B	Increase or toughen the punishment or fines for violators	2 → Poor	3.00	1.44
Q33C	More programs to increase awareness of the young motorists	3 → Fair	2.97	1.44
Q33D	Award system for good drivers without violations	4 → Useful	3.08	1.49
Q33E	Increase or toughening the procedure to get a driver license (training and exam)	5 → Very useful	2.84	1.39
Q33F	Revoking the license of frequent violators		3.22	1.57

## Analysis

### Survey validation using explanatory factor analysis

To validate the survey questionnaire, an explanatory factor analysis (EFA) was conducted. EFA was used to identify the number and nature of the unobserved constructs (latent variables) that are responsible for covariation in the survey responses. In addition, it shows how far the survey was successful in quantifying and measuring the factors affecting traffic safety. The EFA was used as an initial indication for the latent variables that should be used to construct the confirmatory factor analysis (CFA) and the structural equation model (SEM) in this study.

Multiple trials were carried out to achieve the final latent factors to avoid over factored variables and/ or uninterpretable factors. The Kaiser-Meyer-Olkin value (KMO) was found to be 0.791, which is a measure of sample adequacy. A KMO value above 0.5 is considered acceptable, as it indicates that the data was well-factored. Generalized least squares (GLS) was the extraction method considered for the analysis. GLS weights correlation coefficients differentially and treats highly communal variables as more important variables providing better data fitting. A total number of 24 variables were used for the EFA. It is worth mentioning that a range of 20 to 30 variables is adequate to conduct SEM analysis [46]. Table 3 shows the description and measurement scale of the variables used in the analysis.

**Table 3:** Description, codes, and simple statistics for the variables used in the EFA analysis.

Observed Variables		Coding and description of the input value	Simple Statistics	
No.	Description		Mean	SD
Q10	Number of traffic crashes	Integers	0.88	1.40
Q13	Number of traffic violations		0.97	1.61
Q14	Fasten seat belt while driving	1 → Never	3.61	1.36
Q15	Become angry because of another driver and decided to chase him/her	2 → Rarely	3.49	1.27
Q16	Drive at a speed higher than the speed limit		3.28	1.20
Q17	Drive too close to other vehicles (narrow gap)	3 → Sometimes	3.43	1.26
Q18	Change lanes at the last part of a discontinued lane		3.13	1.24
Q19	Run red light when there is no RLR camera, no traffic, or late at night	4 → Often	3.91	1.25
Q20	Drive in the opposite direction		3.75	1.31
Q21	Pass the leading vehicle even if driving at the speed limit	5 → Always	3.04	1.29
Q22	Adjust radio / CD while driving		2.78	1.37
Q23	Cross the intersection at the beginning of a red-light phase		3.61	1.32
Q24	Smoke, eat, or drink while driving		3.10	1.27
Q25	Cell phone use while driving in the case of clear weather		3.18	1.25
Q26	Cell phone use while driving in the case of adverse weather (low visibility)		3.72	1.18
Q27	Participate in illegal races with other drivers		3.79	1.33
Q28	Perceived probability of having a crash	1 → Strongly disagree	2.64	1.22
Q29	Perceived dangers of driving	2 → Disagree	2.91	1.16
		3 → Moderate		
Q30	Exceeding the speed limit is acceptable to become first inline	4 → Agree	2.74	1.29
Q31	Exceeding the speed limit is acceptable when the weather conditions are good, and the traffic police is not present	5 → Strongly agree	2.83	1.29
Q32	Method of using the cell phone while driving	1 → Handheld	1.59	0.79
		2 → Headset		
		3 → Silent driving mode		
Q33A	Increase the presence of traffic police and enforcement	1 → Very poor	2.97	1.37
		2 → Poor		
Q33B	Increasing the cost of violations and fines		3.00	1.44
		3 → Fair		
		4 → Useful		
Q33E	Provide a restricted and tough procedure to get a driver license to increase traffic safety for young drivers		2.84	1.39
		5 → Very useful		

A total of five interpretable factors shown in Table 4 were achieved using a cutoff for the factor loading of 0.4 with Varimax orthogonal rotation [46, 47]. The first construct expresses the dangerous driving behavior, in which six questions/variables were loaded into it. The second construct expresses the distraction resulting from secondary tasks performed while driving. The factor was named in-vehicle distraction, where five variables were loaded into it. Rash driving was the third obtained factor with five variables forming it. Three variables were loaded into the fourth factor, which was considered as the crash risk probability. The fifth and last factor was related to law enforcement, in which three variables measured this latent variable.

**Table 4:** EFA results and the obtained constructs.

Variable / Question		Factor Loading				
Seat belt usage_Q14		0.612				
Angry and chase_Q15		0.507				
Run redlight_Q19		0.743				
Wrong-way driving_Q20		0.621				
Cross at the beginning of a red light phase_Q23		0.478				
Involvement in illegal races_Q27		0.493				
Radio usage_Q22			0.516			
Smoke eat drink while driving_Q24			0.671			
Cell phone usage in clear weather_Q25			0.758			
Cell phone usage in adverse weather_Q26			0.400			
Method of using cell phone _Q32			0.551			
Drive above the speed limit_Q16				0.499		
Drive close to other vehicles_Q17				0.433		
Pass drivers on speed limit_Q21				0.509		
Speed to be first_Q30				0.760		
Speed when no surveillance_Q31				0.805		
Number of crashes_Q10					0.507	
Number of violations_Q13					0.411	
Perceived probability of having a crash_Q28					0.822	
Police presence_Q33A						0.740
Increase the cost of violations _Q33B						0.401
Hard driving license exams_Q33E						0.465
# of factor	Construct	Question #				
Factor #1	Dangerous driving behavior	Q(14, 15, 19, 20, 23, and 27)				
Factor #2	In-vehicle distractions	Q(22, 24, 25, 26, and 32)				
Factor #3	Rash driving behavior	Q(16, 17, 21, 30, and 31)				
Factor #4	Crash risk probability	Q(10, 13, and 28)				
Factor #5	Law enforcement	Q33(A, B, and E)				

As mentioned earlier, one of the main objectives of conducting this study was to investigate the factors that might increase crash risk. The six obtained constructs from the EFA succeeded in explaining the main context of the survey.

### Structural Equation Modeling (SEM)

Structural equation modeling (SEM) is a statistical technique, which can process endogenous and exogenous variables to identify the directional relationships between observed and/or latent variables. Confirmatory factor analysis (CFA) and path model analysis are the two main components of SEM, where simultaneous equations are formed by linking the variables in the model [48]. SEM analysis is commonly used to analyze social sciences datasets. Transportation researchers have recently adopted the SEM to analyze driving behavior questionnaires [47–51]. SEM is deemed a large sample technique, where hypotheses about the means, variances, and covariances of observed data are defined by a hypothesized underlying model [30, 52]

The SEM was conducted in this research using the covariance analysis of linear structural equations (CALIS) procedure of SAS® software (version 9.4). CFA is the first step to conduct the SEM as it provides indications about the latent variables that could be used to develop the path model. Although it explains the relationship between the observed and latent variables, it does not find any causal relationships between the latent variables. In the path model analysis, which is the second step in the SEM, the model path is modified to investigate the direct relationships between the latent variables producing a causal model. Eqs 1 and 2 show the measurement and structural model used in this study [53].

$$v_{i}=\lambda_{i}F_{i}+e_{i}$$ (1)

$$F_{i}^{**}=B_{i}F_{i}^{*}+\Gamma_{i}F_{i}+r_{i}$$ (2)

Where:

v_i_: Vector of observed variables,

λ_i_: Vector of parameters,

F_i_: Vector of latent constructs,

e_i_: Vector of measurement errors,

$F_{i}^{**}:$ Endogenous variables,

B_i_: Parameter vector,

$F_{i}^{*}:$ Mediating variables,

Γ_i_: Parameter vector,

F_i_: Exogenous variables, and

r_i_: Residuals term.

It is worth mentioning that to develop a SEM that is intelligible and practical, several path models have been tested. Engineering judgment and goodness of fit for the model were the two measures used to determine the optimum path model.

### SEM results

Byrne stated that it is preferable to have three indicator variables or more factored in each construct to avoid identification and convergence problems [49]. O'Rourke and Hatcher also recommended having a total number of indicator variables that is less than 30 to avoid the inability to fitting the model [54]. Considering the previously mentioned limitations, a final SEM path was achieved by investigating several SEM paths. The crash risk probability latent factor was used as the outcome of the other constructs. Among the other four latent variables, only three were found to be significant in the path model, with a total number of 17 indicator variables.

**Fig 1:**
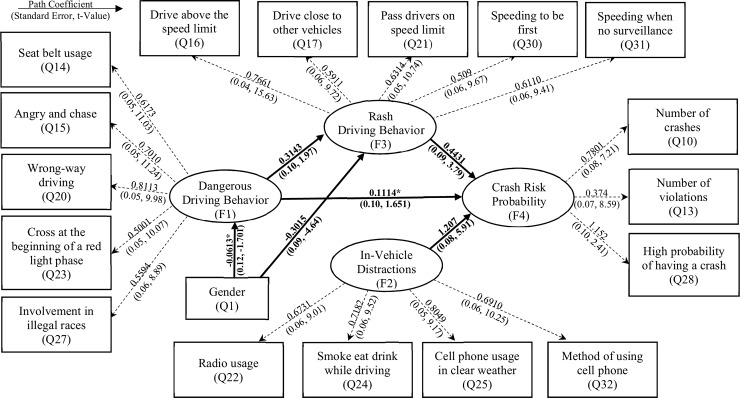
Developed path diagram, path coefficients, and the standard errors for the SEM.

Engineering, education, and enforcement are the three mandatory E’s for road safety. Three indicator variables were factored to form the enforcement latent variable. However, when developing the path model, enforcement was not found to be a significant latent variable in quantifying crash risk. This result might be due to the lack of the other two E’s affecting road safety. Due to the nature of the study, which investigates the crash risk among young drivers, there was no variability in education level and years of driving experience. Nearly 75% of the participants were males, and 25% were females, which represents the gender distribution in Qatar. The gender was used as an indicator variable in the SEM analysis to understand the difference in behavior between males and females. The developed path diagram, path coefficients, and the standard errors for the SEM are shown in Fig 1.

Rash driving behavior was considered to be an endogenous mediator variable. It had a direct effect on crash risk probability with a path coefficient equal to 0.4431. Speeding is considered one of the main exposures that increase crash risk probability [51, 52, 55]. Dangerous driving behavior was found to have a direct effect on rash driving behavior and crash risk probability latent variables with a path coefficient of 0.3143 and 0.1114, respectively. It should be mentioned that the path between the crash risk probability and the dangerous driving behavior was significant at a 90% significance level. The obtained results were in agreement with the literature [30, 56, 57]. In-vehicle distractions were found to be the most significant latent variable that affects the crash risk probability. It had a path coefficient of 1.207, with the highest t-value of 5.91.

The gender was investigated to see how it would affect the crash risk probability. The results indicated that gender had a direct effect on dangerous driving behavior and rash driving. The relationship between the gender and the dangerous driving behavior was found to be significant at a 90% significance level, with a path coefficient of -0.0613. Moreover, gender affects the rash driving behavior with a path coefficient of -0.3015. These relationships indicated that gender had an indirect effect on crash risk probability. The negative sign in the path coefficients for the gender shows that males are more risk-takers compared to females, and they are more subjected to high crash risk. This result might be due to the more aggressive drivers male are than females, which is consistent with the literature [58].

### Goodness of fit for SEM

Hooper et al. introduced different guidelines to find the model fit for SEM [59]. The authors indicated that there is a golden rule for the assessment of model fit. Nevertheless, reporting several commonly used indices to assess SEM model fit is used as each index reflects a different aspect of model fit. Table 5 shows the different indices used to evaluate the model fit and the threshold for each index. The Akaike information criterion (AIC) was also used in estimating the best model. AIC is a comparative measure of fit between more than one model. A lower AIC value indicates a better fit model. Also, the goodness of fit index (GFI) is at the threshold, which indicated a good model fit. Moreover, the comparative fit index (CFI) indicates a good model fit with a value of 0.903. The adjusted GFI value was nearly equal to 0.901, which also indicates a good fit. The standardized root mean squared residuals was 0.051. Hu et al. mentioned that a value below 0.08 for the SRMR is used to conclude a good model fit [54]. Finally, the root mean square error of approximation (RMSEA) in the SEM was found to be 0.49, which is within the criteria threshold.

**Table 5:** Summary of model fit indices.

Model Fit Index	Obtained Values	Threshold Values
Standardized RMR (SRMR)	0.051	<0.05
Goodness of Fit Index (GFI)	0.901	>0.9
Parsimony Index—Adjusted GFI (AGFI)	0.898	>0.9
RMSEA Estimate	0.049	<0.05
Akaike Information Criterion (AIC)	409.318	Lower is better
Bentler Comparative Fit Index (CFI)	0.903	>0.9

## Conclusion

Driver distraction is one of the main causes of crashes and has positive effects on the injury severity of drivers [60–62]. The main purpose of this study was to identify and assess the different types and levels of distracted driving among young drivers in Qatar using a survey questionnaire. Unlike other studies, this study went beyond an exclusive focus on driver distractions related to cell phone use to investigate all types of distractions among young drivers, possible interactions between them, and their associated risks. An EFA was conducted to validate the survey questionnaire. The analysis revealed five contributing latent variables: dangerous driving behavior, in-vehicle distractions, rash driving behavior, enforcement, and crash risk.

An SEM analysis was performed to determine the causality between the latent and the indicator variables. The results showed that the most significant latent variable affecting the risk of a crash was in-vehicle distractions. Moreover, rash driving and dangerous driving had a direct effect on crash risk probability. Additionally, dangerous driving had a direct effect on rash driving. Furthermore, the results suggested that females are safer drivers compared to males.

Holding a cell phone while driving was found to have the most significant effect as an in-vehicle distraction, increasing the crash risk. Dangerous driving behavior indirectly affects crash probability, as it affects driving speed. Road rage and/or aggressive driving were found to be the most dangerous driving behavior. The dangerous driving behavior latent variable explains why young people in Qatar drive at high speeds.

Enforcement, as a latent variable, did not have a significant effect on crash risk. However, to improve road safety in Qatar, speed limits should be enforced. Speed could be controlled by combining multiple elements. Launching educational campaigns, modifying roadway designs (adding speed bumps and adopting road diets), or introducing more operational restrictions (speed radars and more police patrols) could be possible solutions. Administering harsher punishments and improving automated monitoring and reporting systems could be additional measures. These types of enforcement systems are considered effective among drivers [63].

Road safety campaigns should be conducted to educate young drivers about the risk associated with disregarding road safety regulations and the importance of not exceeding speed limits, using seat belts, keeping safe distances between vehicles, and avoiding dangerous behaviors such as aggressive maneuvers, driving in the opposite direction, or chasing after other drivers. Training programs focusing on distracted driving and speeding can be effective in changing young drivers’ behavior [64]. Previous studies have shown that drivers regularly underestimate the risks associated with performing various tasks inside the vehicle [36, 37]. Safety campaigns can contribute to raising young drivers’ awareness of these risks.

The main limitation of this study is related to the questions included in the survey questionnaire. Although the questions were designed to eliminate instrument bias (leading questions, loaded questions, negative questions, unstated criteria, etc.) [65], and effort was made to control response bias, the possibility of a certain degree of social desirability bias introduced in the survey cannot be excluded. In addition, the findings pertaining to young drivers may vary between studies, as the definition of a young driver itself varies. This study included drivers in the 18–25 age group due to the minimum driving age in Qatar, while other studies have other age groups, including 16–24 [66], 17–24 [67], and 17–25 [68]. Furthermore, in addition to the factors identified, some other factors related to the driving environment may also have significant effects on driving behavior and crash risk such as roadway network patterns and time of day [69, 70].

## References

[1] Organization, W.H., Global status report on road safety 2015. 2015: World Health Organization.

[2] Violence, W.H.O., I. Prevention, and W.H. Organization, Global status report on road safety 2013: supporting a decade of action. 2013: World Health Organization.

[3] Organization, W.H., Global status report on road safety 2018: Summary. 2018, World Health Organization.

[4] Gonzales M.M., et al, Student drivers: a study of fatal motor vehicle crashes involving 16-year-old drivers. Annals of emergency medicine, 2005 45(2): p. 140–146. 10.1016/j.annemergmed.2004.08.039 15671969

[5] Jones S., A88 Young driver crash rates in Great Britain: trends and comparisons between countries. Journal of Transport & Health, 2015 2(2): p. S51.

[6] Jones S., A89 Girls crash too: trends and comparisons between male and female young driver crash rates in Great Britain. Journal of Transport & Health, 2015 2(2): p. S51–S52.

[7] Harbeck E.L., Glendon A.I., and Hine T.J., Young driver perceived risk and risky driving: A theoretical approach to the “fatal five”. Transportation research part F: traffic psychology and behaviour, 2018 58: p. 392–404.

[8] Shen S. and Neyens D.M., Factors affecting teen drivers' crash-related length of stay in the hospital. Journal of Transport & Health, 2016.

[9] Administration, N.H.T.S., Distracted driving 2011. Traffic Safety Facts Res. Note, DOT HS, 2013 811: p. 737.

[10] Wen H., et al, Effect of music listening on physiological condition, mental workload, and driving performance with consideration of driver temperament. International journal of environmental research and public health, 2019 16(15): p. 2766. 10.3390/ijerph16152766 PMC6695829 31382474

[11] Prat F., et al, Driving distractions: An insight gained from roadside interviews on their prevalence and factors associated with driver distraction. Transportation research part F: traffic psychology and behaviour, 2017 45: p. 194–207.

[12] Strayer D.L., et al, Measuring cognitive distraction in the automobile. 2013.

[13] Lansdown T.C., The temptation to text when driving–Many young drivers just can't resist. Transportation research part F: traffic psychology and behaviour, 2019 65: p. 79–88.

[14] Brown P.M., George A.M., and Rickwood D., Perceived risk and anticipated regret as factors predicting intentions to text while driving among young adults. Transportation research part F: traffic psychology and behaviour, 2019.

[15] Shevlin B.R. and Goodwin K.A., Past behavior and the decision to text while driving among young adults. Transportation research part F: traffic psychology and behaviour, 2019 60: p. 58–67.

[16] Yannis G., et al, Impact of texting on young drivers' behavior and safety on urban and rural roads through a simulation experiment. Journal of safety research, 2014 49: p. 25. e1–31. 24913482 10.1016/j.jsr.2014.02.008

[17] McKeeverJ.D., et al, Driver performance while texting: even a little is too much. Traffic injury prevention, 2013 14(2): p. 132–137. 10.1080/15389588.2012.699695 23343021

[18] Zicat E., et al, Cognitive function and young drivers: The relationship between driving, attitudes, personality and cognition. Transportation research part F: traffic psychology and behaviour, 2018 55: p. 341–352.

[19] Bendak S., Objective assessment of the effects of texting while driving: a simulator study. International journal of injury control and safety promotion, 2015 22(4): p. 387–392. 10.1080/17457300.2014.942325 25084803

[20] Stavrinos D., et al, Impact of distracted driving on safety and traffic flow. Accident Analysis & Prevention, 2013 61: p. 63–70. 23465745 10.1016/j.aap.2013.02.003 PMC4435680

[21] Caird J.K., et al, A meta-analysis of the effects of texting on driving. Accident Analysis & Prevention, 2014 71: p. 311–318. 24983189 10.1016/j.aap.2014.06.005

[22] Yannis G., et al, P14 Investigating the different distraction mechanism between cell phone use and conversation with the passenger, through a driving simulator experiment. Journal of Transport & Health, 2015 2(2): p. S70–S71.

[23] Papantoniou P., et al, P10 How cell phone use affects reaction time of older drivers. Journal of Transport & Health, 2015 2(2): p. S68–S69.

[24] Ortiz N., Ramnarayan M., and Mizenko K., P08-The Effect of Distraction on Road User Behavior: An Observational Pilot Study Across Intersections in Washington, DC. Journal of Transport & Health, 2016 3(2): p. S67.

[25] McEvoy S.P., Stevenson M.R., and Woodward M., The prevalence of, and factors associated with, serious crashes involving a distracting activity. Accident Analysis & Prevention, 2007 39(3): p. 475–482. 10.1016/j.aap.2006.09.005 17034748

[26] Klauer S.G., et al, The impact of driver inattention on near-crash/crash risk: An analysis using the 100-car naturalistic driving study data. 2006.

[27] Stutts J.C. and Hunter W.W., Driver inattention, driver distraction and traffic crashes. ITE journal, 2003 73(7): p. 34–45.

[28] Klauer S.G., et al, Distracted driving and risk of road crashes among novice and experienced drivers. New England journal of medicine, 2014 370(1): p. 54–59. 10.1056/NEJMsa1204142 24382065 PMC4183154

[29] Shaaban K. and Abdelwarith K., Understanding the association between cell phone use while driving and seat belt noncompliance in Qatar using logit models. Journal of Transportation Safety & Security, 2018 10.4271/2016-01-1439 27648455 PMC5026383

[30] Shaaban K., Gaweesh S., and Ahmed M., Characteristics and Mitigation Strategies for Cell Phone Use While Driving Among Young Drivers in Qatar. Journal of Transport & Health, 2018.

[31] Yannis G., et al, Simulation of texting impact on young drivers’ behavior and safety on motorways. Transportation research part F: traffic psychology and behaviour, 2016 41: p. 10–18.

[32] Cazzulino F., et al, Cell phones and young drivers: a systematic review regarding the association between psychological factors and prevention. Traffic injury prevention, 2014 15(3): p. 234–242. 10.1080/15389588.2013.822075 24372495

[33] Atchley P., Atwood S., and Boulton A., The choice to text and drive in younger drivers: Behavior may shape attitude. Accident Analysis & Prevention, 2011 43(1): p. 134–142. 21094307 10.1016/j.aap.2010.08.003

[34] Tison J., Chaudhary N., and Cosgrove L., National phone survey on distracted driving attitudes and behaviors. 2011.

[35] Horrey W.J., Lesch M.F., and Garabet A., Assessing the awareness of performance decrements in distracted drivers. Accident Analysis & Prevention, 2008 40(2): p. 675–682. 18329420 10.1016/j.aap.2007.09.004

[36] Lesch M.F. and Hancock P.A., Driving performance during concurrent cell-phone use: are drivers aware of their performance decrements? Accident Analysis & Prevention, 2004 36(3): p. 471–480. 15003592 10.1016/S0001-4575(03)00042-3

[37] White M.P., Eiser J.R., and Harris P.R., Risk perceptions of mobile phone use while driving. Risk analysis, 2004 24(2): p. 323–334. 10.1111/j.0272-4332.2004.00434.x 15078303

[38] Walsh S.P., et al, Dialling and driving: Factors influencing intentions to use a mobile phone while driving. Accident Analysis & Prevention, 2008 40(6): p. 1893–1900. 19068291 10.1016/j.aap.2008.07.005

[39] Vanlaar W., Simpson H., and Robertson R., A perceptual map for understanding concern about unsafe driving behaviours. Accident Analysis & Prevention, 2008 40(5): p. 1667–1673. 18760094 10.1016/j.aap.2008.05.009

[40] Shaaban K., Comparative Study of Road Traffic Rules in Qatar Compared to Western Countries. Transport Research Arena, 2012 48: p. 992–999.

[41] Statistical Analysis Office, M.o.I., Ministry of Interior Traffic Accidents Report for the Year 2011. 2 2012.

[42] Shaaban K., et al, Severity analysis of red-light-running-related crashes using structural equation modeling. Journal of Transportation Safety & Security, 2019: p. 1–20. 10.4271/2016-01-1439 27648455 PMC5026383

[43] Shaaban K. and Pande A., Evaluation of Red Light Camera Enforcement Using Traffic Violations. Journal of Traffic and Transportation Engineering (English Edition), 2018.

[44] Shaaban K., Wood J.S., and Gayah V.V., Investigating driver behavior at minor-street stop-controlled intersections in Qatar. Transportation Research Record: Journal of the Transportation Research Board, 2017(2663): p. 109–116.

[45] Bener A., et al, Mobile phone use while driving: a major public health problem in an Arabian society, State of Qatar—mobile phone use and the risk of motor vehicle crashes. Journal of Public Health, 2010 18(2): p. 123–129.

[46] Wong I.Y., Smith S.S., and Sullivan K.A., Validating an older adult driving behaviour model with structural equation modelling and confirmatory factor analysis. Transportation Research Part F: Traffic Psychology and Behaviour, 2017.

[47] Fuller B.T., et al, Ultrafiltration for asphalt removal from bone collagen for radiocarbon dating and isotopic analysis of Pleistocene fauna at the tar pits of Rancho La Brea, Los Angeles, California. Quaternary Geochronology, 2014 22: p. 85–98.

[48] Hassan H.M. and Abdel-Aty M.A., Analysis of drivers’ behavior under reduced visibility conditions using a structural equation modeling approach. Transportation research part F: traffic psychology and behaviour, 2011 14(6): p. 614–625.

[49] Byrne B.M., Structural equation modeling with LISREL, PRELIS, and SIMPLIS: Basic concepts, applications, and programming. 2013: Psychology Press.

[50] Hamed Al Reesi A.A.M., Kai Plankermann, Mustafa Al Hinai, et al, Risky driving behavior among university students and staffin the Sultanate ofOman. Accident Analysis and Prevention, 2013: p. 1–9. 10.1016/j.aap.2013.04.021 23689200

[51] Scott-Parker B., et al, Speeding by young novice drivers: What can personal characteristics and psychosocial theory add to our understanding? Accident Analysis & Prevention, 2013 50: p. 242–250. 22608268 10.1016/j.aap.2012.04.010

[52] De Pelsmacker P. and Janssens W., The effect of norms, attitudes and habits on speeding behavior: Scale development and model building and estimation. Accident Analysis & Prevention, 2007 39(1): p. 6–15. 10.1016/j.aap.2006.05.011 16890180

[53] Kim K., Pant P., and Yamashita E., Measuring influence of accessibility on accident severity with structural equation modeling. Transportation Research Record: Journal of the Transportation Research Board, 2011 2236(1): p. 1–10.

[54] O'Rourke N. and Hatcher L., A step-by-step approach to using SAS for factor analysis and structural equation modeling. 2013: Sas Institute.

[55] Al Reesi H., et al, Measuring risky driving behaviours among young drivers: development of a scale for the Oman setting. Transportation research part F: traffic psychology and behaviour, 2018 55: p. 78–89.

[56] Neyens D.M. and Boyle L.N., The influence of driver distraction on the severity of injuries sustained by teenage drivers and their passengers. Accident Analysis & Prevention, 2008 40(1): p. 254–259. 18215556 10.1016/j.aap.2007.06.005

[57] McEvoy S.P., Stevenson M.R., and Woodward M., The impact of driver distraction on road safety: results from a representative survey in two Australian states. Injury prevention, 2006 12(4): p. 242–247. 10.1136/ip.2006.012336 16887946 PMC2586781

[58] Byrnes J.P., Miller D.C., and Schafer W.D., Gender differences in risk taking: A meta-analysis. Psychological bulletin, 1999 125(3): p. 367.

[59] Hooper D., Coughlan J., and Mullen M., Structural equation modelling: Guidelines for determining model fit. Articles, 2008: p. 2.

[60] Chen F., Song M., and Ma X., Investigation on the injury severity of drivers in rear-end collisions between cars using a random parameters bivariate ordered probit model. International journal of environmental research and public health, 2019 16(14): p. 2632. 10.3390/ijerph16142632 PMC6678079 31340600

[61] Chen F. and Chen S., Injury severities of truck drivers in single-and multi-vehicle accidents on rural highways. Accident Analysis & Prevention, 2011 43(5): p. 1677–1688. 21658494 10.1016/j.aap.2011.03.026

[62] Zeng Q., et al, Investigating the impacts of real-time weather conditions on freeway crash severity: a Bayesian spatial analysis. International journal of environmental research and public health, 2020 17(8): p. 2768. 10.3390/ijerph17082768 PMC7215785 32316427

[63] Shaaban K., Assessment of Drivers' Perceptions of Various Police Enforcement Strategies and Associated Penalties and Rewards. Journal of Advanced Transportation, 2017. 2017: p. 14.

[64] Prabhakharan P. and Molesworth B.R., Repairing faulty scripts to reduce speeding behaviour in young drivers. Accident Analysis & Prevention, 2011 43(5): p. 1696–1702. 21658496 10.1016/j.aap.2011.03.028

[65] Floyd F.J. and Widaman K.F., Factor analysis in the development and refinement of clinical assessment instruments. Psychological assessment, 1995 7(3): p. 286.

[66] Oviedo-Trespalacios O. and Scott-Parker B., The sex disparity in risky driving: A survey of Colombian young drivers. Traffic injury prevention, 2018 19(1): p. 9–17. 10.1080/15389588.2017.1333606 28548584

[67] Ross V., et al, Investigating risky, distracting, and protective peer passenger effects in a dual process framework. Accident Analysis & Prevention, 2016 93: p. 217–225. 27218409 10.1016/j.aap.2016.05.007

[68] Allen S., Murphy K., and Bates L., What drives compliance? The effect of deterrence and shame emotions on young drivers’ compliance with road laws. Policing and society, 2017 27(8): p. 884–898.

[69] Zeng Q., et al, Jointly modeling area-level crash rates by severity: a Bayesian multivariate random-parameters spatio-temporal Tobit regression. Transportmetrica A: Transport Science, 2019 15(2): p. 1867–1884.

[70] Zeng Q., et al, Spatial joint analysis for zonal daytime and nighttime crash frequencies using a Bayesian bivariate conditional autoregressive model. Journal of Transportation Safety & Security, 2018: p. 1–20. 10.4271/2016-01-1439 27648455 PMC5026383

